# CpG Methylation of a Silent Controlling Element in the Murine *A^vy^* Allele Is Incomplete and Unresponsive to Methyl Donor Supplementation

**DOI:** 10.1371/journal.pone.0009055

**Published:** 2010-02-04

**Authors:** Jennifer E. Cropley, Catherine M. Suter, Kenneth B. Beckman, David I. K. Martin

**Affiliations:** 1 Victor Chang Cardiac Research Institute, Darlinghurst, New South Wales, Australia; 2 Faculty of Medicine, University of New South Wales, Kensington, New South Wales, Australia; 3 Children's Hospital Oakland Research Institute, Oakland, California, United States of America; George Washington University, United States of America

## Abstract

**Background:**

The viable yellow allele of *agouti* (*A^vy^*) is remarkable for its unstable and partially heritable epigenetic state, which produces wide variation in phenotypes of isogenic mice. In the *A^vy^* allele an inserted intracisternal A particle (IAP) acts as a controlling element which deregulates expression of *agouti* by transcription from the LTR of the IAP; the phenotypic state has been linked to CpG methylation of the LTR. Phenotypic variation between *A^vy^* mice indicates that the epigenetic state of the IAP is unstable in the germline.

**Principal Findings:**

We have made a detailed examination of somatic methylation of the IAP using bisulphite allelic sequencing, and find that the promoter is incompletely methylated even when it is transcriptionally silent. *In utero* exposure to supplementary methyl donors, which alters the spectrum of *A^vy^* phenotypes, does not increase the density of CpG methylation in the silent LTR.

**Conclusions:**

Our findings suggest that, contrary to previous supposition, methyl donor supplementation acts through an indirect mechanism to silence *A^vy^*. The incomplete cytosine methylation we observe at the somatically silent *A^vy^* allele may reflect its unstable germline state, and the influence of epigenetic modifications underlying CpG methylation.

## Introduction

Retroelements, genomic parasites that amplify via reverse transcription of their RNA and re-insertion into the host genome, make up a large proportion of the DNA of mammalian species [1], [2], [3]. Insertion of retroelements can disrupt genomic structure in several ways [4], [5], but can also disrupt gene regulation [6]. While most retroelements have accumulated mutations that make them unable to retrotranspose, a very large number probably retain intact promoters which, when active, can interfere with transcription of genes in their vicinity [1], [6], [7]. Retroelements are generally maintained in a silent epigenetic state, and carry dense CpG methylation; epigenetic suppression of retroelements is a key function of CpG methylation in vertebrates [8], [9].

Retroelements may escape epigenetic silencing and interfere with transcription of neighbouring genes. An example is the intracisternal A-particle (IAP) retrotransposon responsible for the murine agouti viable yellow alleles, including the *A^vy^* allele that is the subject of this study. In the viable yellow alleles of *agouti*, an IAP is inserted upstream of *agouti* (a different site in each allele) and exhibits a strong propensity for transcriptional activity: it is active in a high proportion of *A^vy^* mice and frequently reverts in the germline from methylated and silent to unmethylated and active [10], [11], [12], [13], [14], [15], [16].

**Figure 1:**
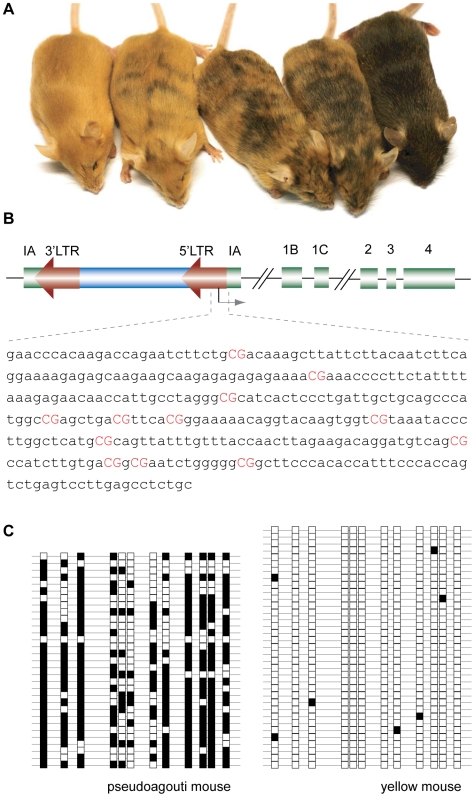
Phenotypic variation in *A^vy^* mice, *A^vy^* allele structure, and complex CpG methylation at the IAP. **A.** Phenotypes of isogenic *A^vy^* littermates range from pure yellow and obese (left) through mottled yellow/agouti to lean fully agouti, called pseudoagouti (right). **B.** Schematic of the *A^vy^* locus, with the sequence of the amplified region, which includes portions of the 5′LTR and pseudoexon 1A (−240 to +92 relative to the cryptic promoter, which is marked by an arrow). The start point of *A^vy^* transcription [11] is marked by an arrow. CpG dinucleotides are displayed in red. **C.** Representative bisulphite allelic sequencing profiles of individual alleles from yellow and pseudoagouti mice. Each single row represents a single allele, and each box a CpG (white: unmethylated; black: methylated).

The labile epigenetic state of the *A^vy^* IAP results in extremely variable penetrance of the associated phenotype in isogenic mice [13], [14]. When the IAP is active, a cryptic promoter in its 5′ LTR usurps transcriptional control of *agouti* and drives ectopic expression of the agouti signalling protein (ASP) [11]. Pancellular expression of ASP produces a neomorphic phenotype of yellow fur, obesity, Type II diabetes, and predisposition to tumors [15]. When the IAP is silent, *agouti* is expressed in its normal hair cycle-specific pattern, giving the wild-type agouti coat colour (called pseudoagouti). The variable activity of the IAP results in a spectrum of phenotypes from fully yellow and obese, through degrees of variegated yellow/agouti with intermediate body mass, to lean pseudoagouti (Figure 1A). *A^vy^* mice always produce offspring with a range of phenotypes, indicating that the epigenetic state of the allele is unstable in the germline [13], [15]. Maternal phenotype does however influence the phenotype of offspring (paternal phenotype does not), consistent with weak inheritance of the epigenetic state through the female germline [13], [15].

The *A^vy^* phenotype appears to correlate with cytosine methylation of the 5′LTR of the IAP: in pseudoagouti mice the LTR is methylated, in yellow mice it is unmethylated, and in mottled mice methylation is intermediate [13], [17], [18], [19]. Dietary supplementation of pregnant dams with methyl donor molecules shifts the phenotype of *A^vy^* offspring towards pseudoagouti, and this effect has been attributed to an increase in methylation at the allele [17], [20]. However, none of these analyses have used methods which provide allelic patterns of methylation at *A^vy^*, so detailed knowledge of *A^vy^* methylation patterns is lacking.

We have used bisulphite allelic sequencing to assess cytosine methylation in the IAP at *A^vy^*, and in three similar IAPs selected from the mouse genome. We find that the relationship between activity state and methylation state at the *A^vy^* allele is not the simple “all-or-none” pattern that has been supposed on the basis of less detailed methylation analyses. The silent *A^vy^* IAP is incompletely methylated in comparison to the three other IAPs, and we speculate that this is related to its germline epigenetic lability. Maternal methyl donor supplementation did not increase the density of cytosine methylation on the silent *A^vy^* IAP. Our results highlight the complex nature of epigenetic regulation at *A^vy^* and support a view of cytosine methylation as a secondary mark of epigenetic silence at *A^vy^*.

## Results

### Cytosine Methylation at *A^vy^* in Yellow and Pseudoagouti Mice

Previous investigations have linked the activity state of the *A^vy^* allele with CpG methylation of the 5′LTR of the inserted IAP, where ectopic transcription of *agouti* is initiated [13], [17], [18], [19]. Results have suggested that in yellow mice the allele is unmethylated, in pseudoagouti mice it is methylated, and in mottled mice the methylation is intermediate; consistent results have been obtained with two other viable yellow alleles [10], [12]. The evidence is derived from Southern blot analysis with methylation-specific restriction enzymes [10], [12], [13], [17], and from sequencing of PCR products amplified from bisulphite-treated DNA in the region of the cryptic promoter in the 5′ LTR [18] or the adjacent pseudoexon 1A [19]. These methods do not provide information on allelic methylation patterns, but instead give an average of methylation on all alleles in the sample population. Studies that have used bisulphite allelic sequencing have not examined the relationship between phenotype and methylation pattern [21], [22]. To analyse allelic patterns of methylation at *A^vy^*, we performed bisulphite allelic sequencing [23] of the *A^vy^* IAP 5′LTR on tail DNA from yellow and pseudoagouti mice. In this method, each sequence is derived from an individual allele in the tissue from which the DNA was extracted. By sequencing large numbers of alleles, and displaying them together, a picture of epiallelic variation in an individual or tissue can be assembled.

As expected, we found that obese yellow mice (in which activity of the IAP is pancellular) had almost no methylation at *A^vy^* (Figure 1C). On the basis of the work noted above, and much evidence that CpG hypermethylation is characteristic of silent retroelements, we expected that pseudoagouti mice would display the opposite pattern: dense CpG methylation. Instead we found that CpG methylation of the IAP in pseudoagouti mice is incomplete (Figure 1C). Very few alleles had methylation of all CpGs in the amplicon, and some alleles did not carry any methylation at all. An average of 66% of CpGs were methylated across all *A^vy^* alleles from pseudoagouti mice. This result was surprising because previous studies examining other silent IAPs had indicated much heavier methylation density at silent LTRs [22], [24], [25].

### The Silent *A^vy^* IAP Is Incompletely Methylated

Dense cytosine methylation is a characteristic feature of vertebrate retroelements, and is linked to their transcriptional silence [8]; thus the incomplete methylation of the silent *A^vy^* IAP would seem to be highly unusual. Another highly unusual feature of the *A^vy^* IAP is its germline epigenetic instability: from one generation to the next, the phenotype is only weakly stable with maternal transmission and completely unstable with paternal transmission [13], [14]. We supposed that incomplete methylation of the *A^vy^* IAP might be related to its germline epigenetic lability. We compared the methylation density in this element with other IAPs in *A^vy^* mice, which do not appear to show epigenetic variation.

We chose from the mouse genome database three IAPs of the same class (IΔ1) as the *A^vy^* IAP, here denoted as “A”, “B” and “C”. (see Methods for details on these elements). Southern blotting of these IAPs with methylation-specific restriction enzymes indicated that they are consistently methylated in *A^vy^* mice of all phenotypes (data not shown). We studied the pattern and density of CpG methylation in the 5′LTRs of these elements with bisulphite allelic sequencing, and compared it with methylation at the *A^vy^* IAP.

**Figure 2:**
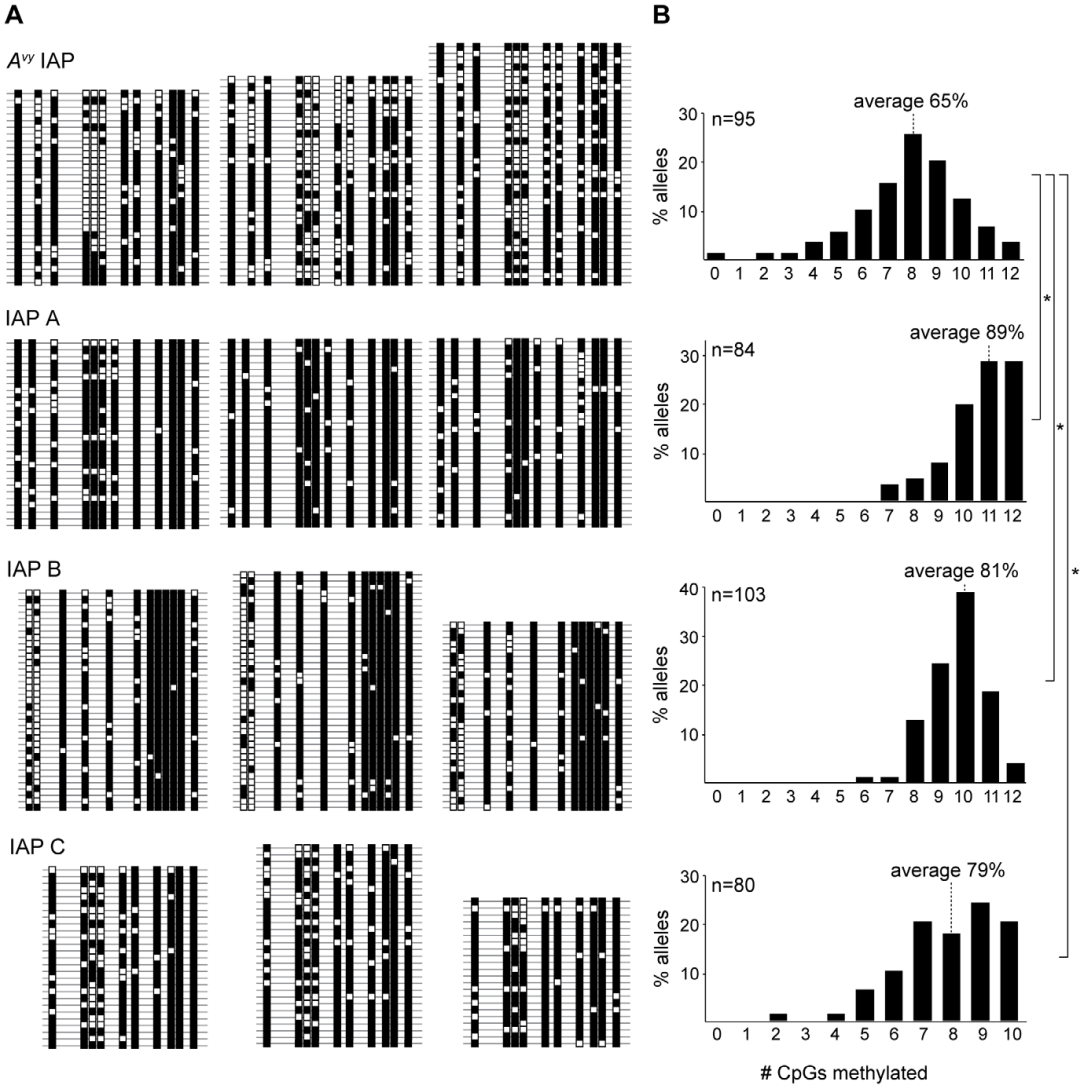
Incomplete CpG methylation of the silent *A^vy^* IAP. **A.** Bisulphite allelic sequencing profiles at the *A^vy^* IAP and three other IAPs. Each line represents an allele, and each box a CpG dinucleotide (white: unmethylated; black: methylated). Each block represents sequences derived from a single mouse. **B.** Histograms of allelic CpG methylation density. Each histogram displays the frequency of alleles with a given number of CpGs methylated across the sequenced region. For IAP C, only 10 CpGs were sequenced. n = number of alleles sequenced; *p<0.001.

All three of the selected IAPs displayed significantly more CpG methylation in their 5′LTRs than did the pseudoagouti (silent) *A^vy^* IAP (Figure 2A). IAP A was the most densely methylated, and IAPs B and C displayed an intermediate methylation density which was still heavier than the methylation at the silent *A^vy^* IAP. In each of the four IAPs methylation density displayed a normal distribution around the mean percent methylation (Figure 2B), but in IAPs A, B and C the mean percent methylation is higher. These results support the view that methylation of the *A^vy^* IAP is relatively incomplete even when it is silent.

### Maternal Methyl-Donor Supplementation Does Not Increase Methylation Density in the Silent *A^vy^* IAP

Dietary supplementation with methyl donors stabilizes the silent state of the *A^vy^* allele. When pregnant dams are fed a diet supplemented with methyl donors, the proportion of pseudoagouti offspring rises [17], [19], [26], [27]. The effect persists into the next generation even when supplementation is carried out only during midgestation, indicating that the methyl donors have affected the germline epigenetic state of *A^vy^* [27]. It has been proposed that methyl donors act on the *A^vy^* allele by increasing methylation of the IAP [17], [19], [26]. As discussed above, we supposed that the incomplete methylation we observed at silent *A^vy^* alleles might be related to its unstable epigenetic state in the germline. We thus asked if the increase in germline epigenetic stability observed after methyl donor supplementation is accompanied by an increase in the density of methylation at the silent *A^vy^*.

We used bisulphite allelic sequencing to assess CpG methylation of the *A^vy^* IAP in pseudoagouti mice that had been supplemented *in utero* with methyl donors (choline, betaine, L-methionine, zinc, folic acid, and vitamin B12; see Methods), and in their unsupplemented pseudoagouti offspring. These were compared with methylation densities in mice that had never received supplementation (and were not descended from supplemented mice). We analysed three mice from each group and pooled the results.

**Figure 3:**
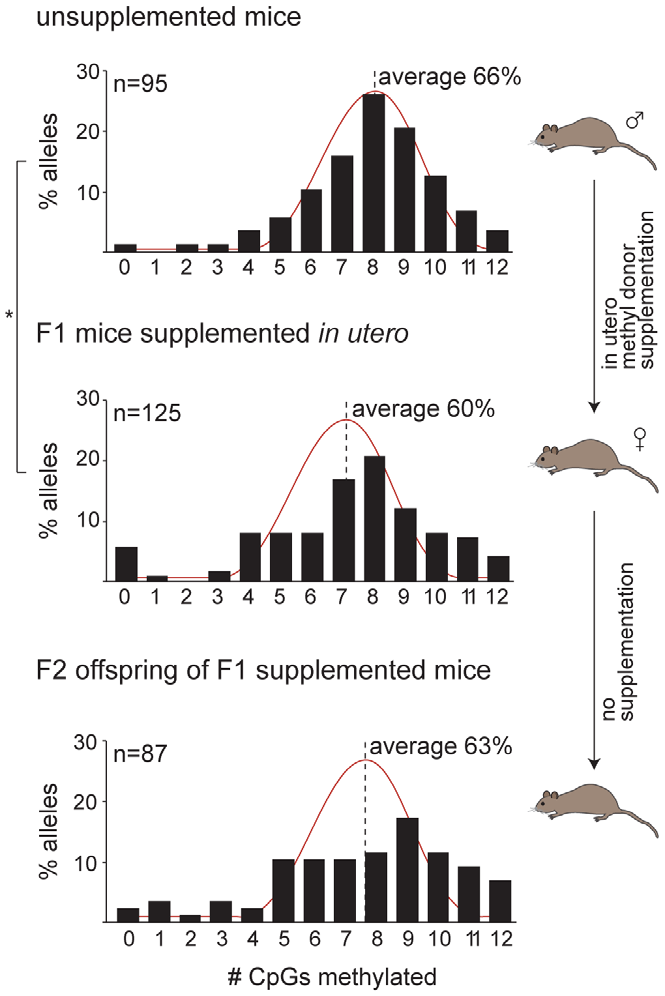
Disruption of cytosine methylation at the silent *A^vy^* allele by methyl-donor supplementation. The histograms display the frequency of alleles with a given number of CpGs methylated across the sequenced region in unsupplemented mice (*top*), mice exposed to methyl-donor supplementation in utero (*middle*) and unsupplemented offspring of supplemented mice (*bottom*). Each histogram represents combined bisulphite allelic sequencing data from three pseudoagouti mice. The red line shows the pattern of normal distribution. The average percent methylation over all sequenced CpGs for each mouse group is indicated. *p = 0.037.

We found that methyl donor supplementation did not increase the density of CpG methylation at the silent *A^vy^* allele; in fact overall methylation was slightly reduced in comparison to unsupplemented controls (Figure 3). Although this result does not answer our question regarding whether cytosine methylation is directly related to germline stability of *A^vy^*, it may shed light on the molecular mechanism of *A^vy^* silencing by methyl donors, as the result is inconsistent with the view that methyl donors act in some direct way to increase CpG methylation.

## Discussion

We have carried out a detailed investigation of the methylation state of the IAP retrotransposon that acts as a controlling element in the *A^vy^* allele, with unexpected results that give insights into epigenetic regulation at this allele. When compared with silent IAPs that are closely related, the IAP in *A^vy^* is incompletely methylated when it is silent (in pseudoagouti mice); this may be a reflection of an unusual germline epigenetic state.

The *A^vy^* IAP is a “controlling element” in the sense the term was used by McClintock, Brink, and others: a transposable element that controls expression of a gene near its insertion site [7]. Controlling elements may be a common feature of higher eukaryotes [6], [28]. Individual *A^vy^* mice exhibit high variation in phenotype because the epigenetic state of the IAP is mosaic, and when the IAP is active it transcribes the *agouti* gene (without any apparent tissue specificity) [11], [15], [29]. This IAP is unusual among retrotransposons: its epigenetic state is not only somatically mosaic, but highly unstable in the germline. The phenotype of individual *A^vy^* mice seems to be determined early in embryogenesis and maintained thereafter, indicating that in somatic cells the epigenetic state is stable. Although germline inheritance of the epigenetic state of *A^vy^* has been established, the inheritance is weak [13]. All of this is consistent with a tendency for epigenetic marks that influence the somatic state of the *A^vy^* allele to fluctuate in the germline.

The incomplete CpG methylation of the IAP in the *A^vy^* allele is further evidence of its unusual epigenetic state. IAPs and retrotransposons are almost invariably silent; extensive evidence shows that they are heavily methylated, except during differentiation of primordial germ cells and in the preimplantation embryo, where partial or complete demethylation may occur [8]. We find that the *A^vy^* IAP, even when it is silent (i.e. in pseudoagouti mice), is significantly less methylated than other closely related IAPs. We speculate that there is a relationship between the incomplete methylation of the *A^vy^* IAP and its germline epigenetic instability: the instability could be caused by the low density of cytosine methylation at *A^vy^*, or conversely, the incomplete methylation could be reflective of an underlying unstable epigenetic state.

We and others have shown that supplementation of the maternal diet with methyl donors can shift the spectrum of *A^vy^* phenotypes toward pseudoagouti [17], [19], [26], [27]; we have recently shown that this effect occurs only when the allele is paternally derived, and that it can alter the germline epigenetic state of *A^vy^* to produce heritable effects on phenotype [27]. We hypothesised that the stabilising effect of methyl donor supplementation on the silent *A^vy^* allele would involve an increase in methylation density, but the findings presented here demonstrate that it did not. It should be noted however that the stabilising effect of methyl donors is not large: the shift in phenotypes towards pseudoagouti is subtle [17], [19], [26], [27]. It may be that germline silencing of *A^vy^* would have to be very stable before an observable increase in DNA methylation occurred.

However, the fact that CpG methylation at the silent *A^vy^* did not increase in response to methyl donors may provide insight into the mechanism by which methyl donors alter the spectrum of phenotypes in *A^vy^* mice. Methyl donors contribute to the pool of S-adenosylmethionine, which can donate a methyl group to proteins and to cytosine [26]. Previous studies showed that the phenotypic shift induced by methyl donors is accompanied by an increase in CpG methylation at the *A^vy^* allele within the supplemented population: this has been interpreted as indicating that the effect of methyl donors is to directly increase methylation at *A^vy^* and thereby change the phenotype [17], [19]. But the effect of dietary methyl donors could also be mediated by an indirect mechanism, for example by histone modifications [30], [31] in the region of the *A^vy^* allele, resulting in a shift to epigenetic silence. The observed increase in cytosine methylation would thus be due to methylation of the already silenced allele. Our finding favors this latter possibility: the amount of cytosine methylation at the silent *A^vy^* allele is not increased in response to methyl donor supplementation, arguing against a direct effect on CpG methylation. If methyl donors were to act directly on cytosine methylation, one would expect to observe an increase in methylation density not only over the population of supplemented mice as a whole, but also at the extremes: that is, supplemented pseudoagouti mice would show more methylation than pseudoagouti mice that had not been supplemented. Our data show that this is not the case, and suggest that methyl donors silence *A^vy^* through some pathway other than CpG methylation.

Our findings highlight the complex epigenetics of *A^vy^*, and the unusual behaviour of the IAP retrotransposon in this allele. What might distinguish the IAP in the *A^vy^* allele from other retroelements? The *A^vy^* IAP is a relatively recent insertion, arising ∼45 years ago. We propose that most retroelements, once they have inserted into the host genome, undergo a gradual acquisition of suppressive epigenetic marks in the germline; these marks likely include multiple histone modifications as well as CpG methylation and the binding of chromatin proteins. Eventually this accumulation of marks results in a permanently silent state, one that is retained through the mammalian life cycle and is resistant to perturbations such as embryonic demethylation. The process may be similar to the progressive germline silencing observed in some mouse transgenes: the transgenes initially display variable but declining activity, and eventually reach a silent state that is stable for generations; somatic methylation of the transgene is evident only when it has begun the process of germline silencing. One implication of this model is that almost half of the mammalian genome (retroelements) exists in a permanently silent state like that of constitutive heterochromatin; the presence of this material may contribute to the apparently spontaneous occurrence of germline epimutations [32], [33], [34], [35] because silent chromatin is known to spread and silence adjacent euchromatin.

Is the *A^vy^* IAP ever likely to become deeply silent and heavily methylated? The relatively short time that the IAP has been present at *A^vy^* may not be sufficient for a retrotransposon to be permanently silenced [36], [37]. Furthermore, there has been selection for the active state of the IAP, because the obese yellow phenotype that is the result of IAP activity was for most of this time the trait of interest, and the epigenetic basis of the phenotypic variation was not at first understood [36], [37]. It may be that the *A^vy^* allele is a case in which a recently inserted retrotransposon, capable of transcriptional activity, has been maintained in an active state by artificial selection for that state.

The incomplete methylation of *A^vy^* may thus reflect its germline epigenetic instability. The finding that methylation state of the IAP does not precisely correlate with its activity is further evidence that in somatic cells CpG methylation is a reflection of other epigenetic factors that control the transcription state.

## Materials and Methods

### Mice and Diets

All animals were handled in strict accordance with good practice as defined by the Office of Laboratory Animal Welfare (NIH, USA), the NHMRC (Australia) Statement on Animal Experimentation, and the requirements of NSW State Government legislation. All animal work was approved by the CHORI IACUC (Assurance number A3631-01) and the St Vincents/Garvan Animal Ethics Committee (REFS: AEC#06/12; AEC#09/12). The *A^vy^* allele arose in the C3H/HeJ strain [36], and was backcrossed into C57BL/6 at the Jackson Laboratory. The mice used in this study are descended from the isogenic C57BL/6 *A^vy^* colony maintained at Oak Ridge National Laboratories and were rederived at the VCCRI in 2001. Methyl donor supplementation was carried out as previously described [27]. Mice were fed *ad libitum* on NIH-31 diet (control) or methyl-donor supplemented NIH-31 (plus (per kg) 15 g choline, 15 g betaine, 7.5 g L-methionine, 150 mg zinc, 15 mg folic acid, 1.5 mg vitamin B_12_) (Specialty Feeds, Glen Forrest, WA, Australia) [17], [26]. Methyl-donor supplementation was started on day 8.5 of gestation and discontinued at day 15.5. Female pseudoagouti (F1) offspring of supplemented mice were bred to produce the F2 generation. Control groups were made up of mice bred in exactly the same way but without any methyl-donor supplementation.

### Retrotransposon Sequences

The sequence of the *A^vy^* IAP was provided by Hugh Morgan, University of Sydney, Australia. IAPs “A”, “B” and “C” were chosen by searching the mouse genome for sequences similar to the *A^vy^* IAP 5′LTR. The 5′LTR of IAP A has 88% sequence identity with the *A^vy^* IAP LTR and is found on chromosome 10 at (5′) 70,254,133-70,254,485 (3′). The IAP B 5′LTR has 87% identity with the *A^vy^* IAP LTR and is found on chromosome X at (5′) 70,187,292-70,187,643 (3′) in intron 3 of the *nsdhl* gene. The IAP C [24] 5′LTR has 96% identity with the *A^vy^* IAP LTR over its 5′ 244 bp (the region analysed in this study) and is divergent at the 3′ 150 bp; it is found on chromosome 2 at (5′) 154179999-154180392 (3′), in intron 6 of the *Cdk5rap1* gene.

### Bisulphite Treatment and Methylation Sequencing of IAPs

DNA was extracted from mouse tail tips. DNA was extracted by digestion with Proteinase K and purification with phenol/chloroform. 2 µg DNA was treated with sodium bisulphite as described elsewhere [38], [39]. The bisulphite-treated DNA was resuspended in 50 µl water and 5 µl was used in PCR. The primers used to amplify the 5′LTRs were: *A^vy^* IAP: gtagaggtttaaggatttagattggtg (fwd), aacccacaaaaccaaaatcttctac (rev) (primers targeted (-) DNA strand); IAP A: tttatggggttagagtgtaagaagtaag (fwd), caaattaccctattataacaaatatatctc (rev); IAP B: gtgaaygttagttyggttattgggttg (fwd), cttacacctttaaaaactaaataacaaatcc (rev); IAP C: gtatatagttaataagtgggtaatggtg (fwd), caaccattacctaaaacacatcactc (rev). PCR products were cloned into pGEM-T and transformed into *E.coli*, and plasmid DNA from individual colonies was sequenced.

### Statistical Analysis

Methylation data was analysed at the level of individual alleles, so that the number of degrees of freedom relates to the number of alleles sequenced (ANOVA was used to confirm that the methylation levels of individual alleles were independent of the mouse from which the alleles derived). Statistical significance was tested using the Student's t-test with α = 0.05.
